# Insulinization: A promising strategy for the treatment of type 2 diabetes mellitus

**DOI:** 10.3892/etm.2013.1300

**Published:** 2013-09-13

**Authors:** GUO LIAN, XU YUE, ZHANG XIANXIANG, LUO YONG, LIU WEIJUAN, CHEN BING

**Affiliations:** 1 Departments of Endocrinology, Chongqing Three Gorges Central Hospital, Chongqing 404000;; 2 Emergency, Chongqing Three Gorges Central Hospital, Chongqing 404000;; 3 Department of Endocrinology, Southwest Hospital, Third Military Medical University, Chongqing 400038, P.R. China

**Keywords:** insulinization, type 2 diabetes, continuous subcutaneous insulin infusion

## Abstract

The aim of the present study was to assess the efficiency of the long-term use of continuous subcutaneous insulin infusion (CSII), a novel regimen known as insulinization, for the treatment of type 2 diabetes mellitus. A total of 150 subjects who fulfilled the diagnostic criteria for type 2 diabetes were included in the study. The patients were divided into eight groups according to the treatment regimens they received and were monitored for 3 months. Insulin doses were adjusted to optimize glycemic control with the simplest possible insulin regimen. The outcomes studied included the time required for glycemic control and insulin dose reduction, the total daily insulin dose, the ratio of patients not requiring the administration of oral antidiabetic drugs (OADs), the rate of hypoglycemia, and hemoglobin A1c (HbA1c) and fasting plasma insulin (FinS) levels. Patients receiving insulinization required less time to achieve optimal glycemic control and insulin decrement compared with patients receiving other treatments. The total daily insulin dose for patients receiving insulinization therapy was 0.23±0.07 U/kg/day, which was lower than that in any other group. In patients receiving insulinization, the ratio of patients that did not require OADs (43.3%) and the concentration of FinS A were higher than those in the other groups. Furthermore, insulinization resulted in a greater reduction in HbA1c levels, as well as a reduced incidence of severe hypoglycemia. Insulinization may mimic physiological insulin secretion more effectively than other therapies. This regimen is more efficient and reduces the incidence of hypoglycemia in patients with type 2 diabetes, indicating that it is likely to be a promising treatment strategy for the disease.

## Introduction

Diabetes is a common chronic medical condition that leads to increased blood glucose levels (1). Type 2 diabetes, also known as ‘adult-onset’ diabetes, is more common than type 1 diabetes. It is characterized by progressive β-cell dysfunction on a background of peripheral insulin resistance and a defective incretin system (2,3). Although traditionally considered an adult disease, type 2 diabetes is becoming increasingly common in children and teenagers due to an increase in obesity in young people (4). In 2003, it was estimated that there were 150 million individuals globally with type 2 diabetes and that by 2025, this number would reach ~300 million (5). This high prevalence is likely to cause a heavy socio-economic burden. Thus, it is imperative that effective and practical preventative and therapeutic strategies are developed for this disease in order to reduce its rates of morbidity and mortality.

Lifestyle modification is an effective approach for reducing the incidence of type 2 diabetes in at-risk patients (6). However, once the disease is established, sequential addition of medications over time is required in order to maintain adequate glycemic control. Insulin therapy has been integrated into the treatment of type 2 diabetes (7). This treatment paradigm, the earlier initiation of insulin therapy, has been recommended in previous treatment guidelines (8,9). There is evidence to demonstrate that insulin is the antidiabetic agent with the greatest glucose-lowering capacity, and epidemio-logical studies have shown its benefits in terms of improving glycemic control and reducing the risk of long-term diabetic complications in type 1 and type 2 diabetes (10). However, despite these benefits, there are well-documented barriers to insulin therapy, including perceived inconvenience, needle anxiety and low portability of the devices involved (10).

Continuous subcutaneous insulin infusion (CSII) has been shown to offer significant benefits over treatment involving multiple daily insulin injections (MDI) in patients with type 1 diabetes (11,12). Decreased hemoglobin A1c (HbA1c) levels, as well as a decreased incidence of severe hypoglycemia, have been demonstrated in type 1 diabetic patients treated with CSII therapy compared with those receiving MDI therapy (13,14). The benefits of CSII therapy may also be achieved in type 2 diabetic patients who require intensive insulin therapy but seek an alternative to MDI therapy (15,16). These patients have demonstrated similar reductions in HbA1c levels and, in certain cases, have shown improved postprandial glucose control and patient satisfaction with pump use (15). Furthermore, the short-term use of CSII therapy has aided glycemic control in type 2 diabetic patients who failed to maintain glycemic control with diet or oral antidiabetic drugs (OADs) (17). The pump is removed and OADs prescribed once glycemic values are stabilized. However, to the best of our knowledge, no previous studies have evaluated the long-term use of CSII therapy for the treatment of type 2 diabetes. Whether the long-term or short-term use of CSII therapy regimen is more efficient also remains unknown. The aim of the present study was to assess the effects of the long-term use of CSII therapy, termed insulinization, on glycemic control, HbA1c levels and β-cell function. The results may be useful in clinical practice and may introduce a new strategy for the treatment of type 2 diabetes mellitus.

## Materials and methods

### Study subjects

A total of 150 subjects (72 males and 78 females) were enrolled in this study based on the following criteria: i) fulfilled the 1999 WHO type 2 diabetes criteria (T2DM criteria) (5); ii) possessed no severe dysfunction of the heart, brain, lungs, liver or kidneys; iii) not affected by severe stressors, including infection, surgery, trauma or an acute metabolism disorder; iv) not taking additional drugs that interfere with the effects of insulin, for example, high doses of glucocorticoid; v) not pregnant or lactating; and vi) not suffering from diseases of the blood system, malignancy or psychonosema. The study was approved by the Chongqing Three Gorges Central Hospital Ethics Committee (Chongqing, China) and written informed consent was obtained from all participants.

Among the T2DM patients, the average age was 54±1.5 years (range, 34–67 years), the average disease duration was 9±2.5 years (range, 1–20 years) and the average level of HbAlc was 11±1.2% (range, 8.5–15%). There were 57, 55, 68 and 74 patients suffering from peripheral neuropathy (PNP), retinopathy, nephropathy and peripheral arterial disease (PAD), respectively. The numbers of patients that were affected by hypertension, coronary heart disease (CHD), cerebral infarction, dyslipidemia, hypertension and CHD, hypertension and cerebral infarction and CHD and cerebral infarction were 33, 25, 10, 48, 16, 13 and 12, respectively.

### Materials and biochemical measurements

Insulin aspart, insulin aspart 30 and Novolin 30R were purchased from Novo Nordisk (Copenhagen, Denmark). Insulin lispro, insulin lispro 25 and Humulin 70/30 were obtained from Eli Lilly & Co. (Indianapolis, IN, USA). Insulin glargine was purchased from Sanofi-Aventis (Paris, France). Blood sugar was detected using the One Touch Ultra meter (Johnson & Johnson, Zwick, NJ, USA) and HbA1c was detected by HPLC-723 G7 (Bio-Rad, Hercules, CA, USA). Chemiluminescence was used to measure insulin levels. CSII was performed using Medtronic 712 insulin pumps (Medtronic, Minneapolis, MN, USA) filled with insulin aspart.

Fasting glucose levels of 3.5–16 mmol/l or plasma glucose levels of 15–40 mmol/l two hours after a 75-g oral glucose load were considered to be normal values. In the present study, the aim was to control blood glucose levels at 4.4–6.1 mmol/l and 4.4–8.0 mmol/l prior to and after a meal, respectively.

### Experimental design

A total of 150 subjects were randomly divided into five experimental groups (30 patients per group). All patients were monitored for three months. The study protocol and treatments for each group are described below (Table I).

Experiment 1 involved the long-term use of CSII therapy (LC or insulinization group). In this group, the pump was kept in the body after the blood glucose levels had been controlled to normal values by CSII therapy. When the blood glucose levels were reduced to normal values, the injected dose was downregulated, based on the blood glucose level, to the lowest possible dosage at which the blood glucose level was stable. The blood glucose levels were maintained at 4.4–6.1 mmol/l and 4.4–8.0 mmol/l prior to and after a meal, respectively. The subsequent regimen depended on the total daily insulin dose. If the total daily insulin dose was ≥0.7 U/kg, insulin glargine plus insulin aspart (or insulin lispro) was subcutaneously injected. If the total daily insulin dose was ≥0.3 U/kg, insulin aspart 30 or insulin lispro 25 was injected subcutaneously in graded doses. If the total daily insulin dose was ≥0.1 U/kg, antidiabetic drugs were terminated and alternative management strategies, including diet and exercise, were adopted.

Experiment 2 evaluated the short-term use of CSII therapy (SC group). In this group, the insulin pump was removed when blood glucose levels were controlled to normal values by CSII. The regimen adopted subsequently depended on the dosage in the pump used over a whole day. If the total daily insulin dose was ≥0.7 U/kg, insulin glargine plus insulin aspart (or insulin lispro) was subcutaneously injected. If the total daily insulin dose was ≥0.3 U/kg, insulin aspart 30 or insulin lispro 25 was injected subcutaneously. If the total daily insulin dose was ≥0.1 U/kg, antidiabetic drugs were terminated and alternative management strategies, including diet and exercise, were adopted.

Experiment 3 investigated basal plus prandial insulin intensive treatment. Half of the patients were prescribed insulin glargine plus insulin aspart (GA group) and the remainder of the patients were prescribed insulin glargine plus insulin lispro (GL group). When blood glucose was controlled to normal values, the subsequent regimen was adopted according to the dosage used in a whole day. If the total daily insulin dose was ≥0.3 U/kg, the present strategy was maintained. If the total daily insulin dose was <0.3 U/kg, antidiabetic drugs (oral) were administered and complications were treated according to individual conditions. If the total daily insulin dose was <0.1 U/kg, antidiabetic drugs were terminated and management strategies, including diet and exercise, were adopted.

Experiment 4 analyzed intensive treatment with human insulin analogs. Either insulin aspart 30 (IA group, n=15) or insulin lispro 25 (IL group, n=15) was injected into patients subcutaneously three times per day. When blood glucose was controlled to normal values, the subsequent regimen was adopted according to the dosage used in a whole day. If the total daily insulin dose was ≥0.3 U/kg, the initial strategy was continued. If the total daily insulin dose was <0.3 U/kg, antidiabetic drugs were administered orally and complications were treated according to the condition of the individual. If the total daily insulin dose was <0.1 U/kg, antidiabetic drugs were terminated and alternative management strategies, including diet and exercise, were adopted.

Experiment 5 evaluated intensive treatment with human insulin. In this group, Novolin 30R or Humulin 70/30 was injected subcutaneously twice per day. Half of the patients were prescribed Novolin 30R (Nov group) and the remaining patients were prescribed Humulin 70/30 (Hum group).

### Outcome measures

The primary outcome was the assessment of glycemic control. This included the time required to achieve normal or near-normal blood glucose values, the total dosage of insulin used before glycemic control was achieved, and the time required to decrease the insulin and the total daily insulin doses. The secondary outcomes were the results of management, including the ratio of patients who did not require administration of OADs, the time course of the disease when administration of OADs was terminated, the general incidence of hypoglycemia and the body weight status. The third outcome was the evaluation of islet cell function, including the levels of HbA1c and fasting plasma insulin (FinS).

### Statistical analysis

Data are presented as the mean ± SD. Comparisons between the two experimental groups were made using the unpaired Student’s t-test. One-way ANOVA combined with post-hoc least significant difference (LSD) analysis was used for the statistical analysis by employing SPSS 10.0 software (SPSS, Inc., Chicago, IL, USA). P<0.05 was considered to indicate a statistically significant difference.

## Results

### Assessment of glycemic control

Data are shown in Table II. For each cohort and for all patients combined, the time required to achieve normal or near-normal blood glucose values was lower in the CSII therapy groups (LC and SC groups) compared with those in the insulin-intensive treatment groups (GA, GL, IA, IL, Nov and Hum groups; P<0.05 vs. the IA, IL, Nov or Hum groups). Although small differences were observed in the time required for glycemic control among the intensive treatment groups, no significant differences were identified. A reduction in the mean total insulin doses required for glycemic control for all patients in the LC group was also observed. However, this reduction was not significant, possibly due to the relatively small number of patients. Subsequently, the time required for a reduction in the insulin dose was evaluated in each group. It was shown that CSII therapy (LC and SC groups) markedly reduced the time required for insulin decrement (P<0.05 vs. IA, IL, Nov or Hum groups). No significant differences were identified between the IA, IL, Nov and Hum groups. Notably, the total daily insulin dose after decrement was markedly lower in the LC group than in other groups (P<0.05 vs. SC, IA, IL, Nov or Hum groups). No significant differences were identified between the other groups.

### Results of management

The data in Table III reveal that 43.3% of patients were able to stop taking OADs at the end of treatment in the LC group. This proportion was significantly higher than that of any other group (P<0.05 vs. the SC, GA, GL, IA, IL, Nov or Hum groups), indicating that the insulinization regimen led to an improved effect on the recruitment of β-cell function. No significant differences were identified in indices including the time course of the disease on termination of OADs and the ratio of actual weight/standard weight, among the groups (Table III). Standard weight = height − 105.

The incidence of patients who had experienced at least one episode of hypoglycemia (expressed as a percentage) was also investigated in this study. As shown in Table III, it was observed that the incidence rates in the LC and SC groups were 6.7 and 13.3%, respectively. These rates were lower than those of the other groups (Table III). Although a small difference was identified in the LC group compared with the SC group, no statistically significant differences were identified. These results suggest that the insulinization regimen provided lower rates of hypoglycemia than any other group.

### Measurement of HbA1c levels and β-cell function

The HbA1c test is currently one of the optimal methods for checking that diabetes is under control. Therefore, in the present study, the levels of HbA1c before and after treatment were compared across the groups. No significant differences were identified in the HbA1c levels between the groups before treatment (Table IV). However, it was observed that HbA1c levels in the LC (5.7±0.2%), GA (6.4±0.3%) and GL (6.2±0.2%) groups were significantly lower than those of the other treated groups (Table IV; P<0.05 vs. the SC, IA, IL, Nov or Hum groups). No statistically significant differences were identified between the SC, IA, IL, Nov and Hum groups.

In order to investigate whether β-cell function was ameliorated by treatment, FinS levels were examined in each group. As shown in Table IV, the FinS levels did not differ among the groups before treatment. However, after treatment, the FinS levels in the LC, GA and GL groups were higher than those of the other groups (P<0.05 vs. the SC, IA, IL, Nov or Hum groups). Furthermore, a significant difference was identified between the LC group and the GA or GL groups (P<0.05 vs. GA or GL groups), while no statistically significant differences were identified between the SC, IA, IL, Nov and Hum groups. These data indicate that the insulinization regimen led to an improved recovery of β-cell function compared with other approaches to treatment.

## Discussion

The present pilot study assessed the efficiency of the long-term use of CSII therapy, a new regimen termed insulinization, for the treatment of type 2 diabetes. A series of groups were investigated in order to compare the effects on management and prognosis. The aim of this study was to gain information about insulinization for the treatment of type 2 diabetes and provide an alternative therapeutic strategy for type 2 diabetic patients. This information, which to the best of our knowledge is not available in the published literature, is important for patient care with the currently available insulin treatment and also serves as an innovation for diabetic treatment.

CSII is the constant, continuous infusion of short-acting insulin, driven by mechanical force and delivered via a needle or soft cannula under the skin. CSII was originally used to treat type 1 diabetic patients (18,19). It is now widely accepted that insulin aspart in CSII may be a safer and more efficacious therapy than MDI therapy for type 2 diabetes (20). Patients with type 2 diabetes may be trained as outpatients to use CSII and have indicated that they prefer CSII to injections, suggesting that pump therapy should be considered when initiating intensive insulin therapy for type 2 diabetes (20,21). In clinical trials, short-term CSII therapy has been adopted to control glycemic levels in type 2 diabetic patients who failed to maintain glycemic control with diet or OADs (17). However, we observed previously that type 2 diabetic patients receiving long-term CSII therapy (insulinization) had better outcomes of treatment and a better prognosis. Therefore, the aim of the present study was to address this observation.

Optimal glycemic control was achieved with insulin treatment in all groups of patients. However, the time required for achieving normal or near-normal values was less in the CSII therapy patients. The time required to reduce the insulin dose in patients with CSII therapy was also lower compared with that of patients receiving other treatments. The improved efficiency of CSII therapy in glycemic control may be a result of it mimicking physiological insulin secretion in an early and timely manner. Following insulin decrement, the mean total daily insulin dose for patients receiving insulinization was 0.23±0.07 U/kg body weight/day. This dose was markedly lower than that of other regimens, including short-term CSII therapy, indicating that insulinization may not only control blood glucose rapidly, but also reduce the total dosage of insulin during the treatment of type 2 diabetes. Alternative management strategies, including lifestyle intervention and medications (OADs), are major methods of type 2 diabetes treatment when glycemic control has achieved normal values (6). Evidence-based guidelines for the comprehensive management of type 2 diabetes mainly focus on lifestyle interventions, lowering other cardiovascular risk factors and maintaining blood glucose levels in the normal range (6,7). In the present study, we observed that 43.3% of patients receiving insulinization treatment no longer required administration of OADs at the end of treatment, and lifestyle interventions were sufficient to maintain normal blood glucose levels. These results indicate that the long-term use of CSII therapy has a better effect on the prognosis of type 2 diabetes than other treatments.

FinS is an indicator of β-cell function (22). It has been hypothesized that in severe, poorly controlled type 2 diabetic patients, diseased β-cells are forced to secrete immature granules, in which the conversion of pro-insulin to insulin is incomplete (23). Therefore, the improvement of glucose-stimulated insulin secretion is associated with the amelioration of hyperglycemia. In the present study, we demonstrated that the concentration of FinS was markedly increased following treatment, particularly following regimens that mimicked physiological insulin secretion (LC, GA and GL groups). HbA1c is a form of hemoglobin that is measured primarily to identify the average plasma glucose concentration over prolonged periods of time. Normal levels of glucose correspond to a normal quantity of HbA1c, and the fraction of HbA1c increases in a predictable manner following average increases in plasma glucose. Thus, it serves as a marker for average blood glucose levels over the months prior to the measurement, and monitoring HbA1c levels in diabetic patients may improve treatment (24). The patients involved in this study all exhibited levels of HbA1c >6.5%, a criterion for the diagnosis of diabetes that is commended by the 2010 American Diabetes Association Standards of Medical Care in Diabetes (25). In the majority of patients, normal or near-normal levels of HbA1c were achieved following insulin treatment. Compared with other treatment methods, insulinization therapy resulted in more efficient control of HbA1c levels. The decline in HbA1c levels may be due to a reduction in preprandial and postprandial glucose concentrations, since a reduction in postprandial glucose and glucose excursions have been associated with reductions in markers of oxidative stress and inflammation. This may be beneficial for the recovery of islet function and reduce the risk of long-term complications (26,27). Thus, combined with the data concerning FinS, it was concluded that regimens that mimic physiological insulin secretion over a long duration (LC, GA and GL groups) are an improved strategy to recruit islet function for diabetic patients.

Accumulated evidence has shown that hypoglycemia is the most important barrier to the intensification of insulin therapy (28). Hypoglycemia is common and may have serious consequences in patients with type 2 diabetes, as well as in those with type 1 diabetes (29,30). CSII therapy leads to a reduced incidence of hypoglycemia. This may be a result of the insulin release in CSII therapy being similar to physiological insulin secretion. Notably, during the present study, the long-term use of CSII therapy led to the lowest rates of hypoglycemia in diabetic patients. Different pump conditions for regulating insulin release may also contribute to different incidence rates of hypoglycemia. Patients receiving insulinization treatment remained in hospital to allow doctors to regulate the level of insulin released by the pump according to the levels of blood glucose in a timely manner. By contrast, patients in other groups regulated their insulin release independently, under the direction of doctors outside the hospital. This approach leads to a delay in regulation as a result of a lack of close monitoring of blood glucose levels and thus an increased incidence of hypoglycemia.

It is widely accepted that diabetes is a chronic, progressive disease (5,31). With the progressive development of type 2 diabetes, patients eventually require insulin in order to bring their condition under control. Thus, the predominant strategy for controlling type 2 diabetes is to attempt to protect β-cells from oxidative injury and recruit β-cell functions (23,26). A number of studies have indicated that earlier initiation of insulin therapy is beneficial for protecting β-cell function (25,32,33). Compared with the other treatment regimens adopted in the present study, insulinization prolongs the imitation of physiological insulin secretion. This regimen is rarely used in the current clinical treatment of type 2 diabetes. The modes of insulin release in the LC, GA and GL groups are close to that of physiological insulin secretion. These therapies have better effects on the recovery of β-cell function and delay the development of the disease. Furthermore, this reduces the dose of insulin used, particularly with the long-term use of CSII. Although the short-term use of CSII therapy also mimics physiological insulin secretion, the relatively short time of treatment is less efficient compared with the longer time of the insulinization regimen. Similar to atropinization in the treatment of organophosphorus intoxication, insulinization therapy produces a further protective effect on β-cell function and thus is beneficial to the final outcome of type 2 diabetes treatment (34).

The limitations of the current study require consideration. No vehicle control group was used; therefore, it was not possible to make definitive comparisons between alternative therapies. Furthermore, the study was relatively small, short-term (3 months) and conducted in a controlled setting and in clinics specializing in diabetes. However, despite these limitations, this study provides useful information for clinical practice and may serve to aid the ongoing development of insulin treatment regimens specifically for patients with type 2 diabetes.

In conclusion, the long-term use of CSII therapy persistently mimics physiological insulin secretion to reduce hyperglycemia-induced oxidative stress and consequently is beneficial to the recovery of islet function. Patients receiving insulinization therapy exhibit improved treatment outcomes and prognoses compared with those receiving other treatment methods. Collectively, this study demonstrates that the long-term use of CSII therapy (insulinization) mimics physiological insulin secretion more effectively than other treatment strategies and provides a promising strategy for the treatment of diabetes mellitus type 2.

## Figures and Tables

**Table I.** Grouping and treatment protocol of type 2 diabetes patients.

Group	Number of cases	Treatment protocol
LC	30	Long-term CSII therapy (insulinization)
SC	30	Short-term CSII therapy
GA	15	Glargine + aspart intensive treatment
GL	15	Glargine + lyspro intensive treatment
IA	15	Insulin aspart 30; three times a day
IL	15	Insulin lispro 25; three times a day
Nov	15	Novolin 30R; two times a day
Hum	15	Humulin 70/30; two times a day

CSII, continuous subcutaneous insulin infusion.

**Table II.** Outcome of the assessment of glycemic control in each group.

Group	Time required for glycemic control (days)	Total insulin doses used before insulin decrement (U/kg/day)	Time needed for insulin decrement (days)	Total daily insulin doses after decrement (U/kg/day)
LC	3.8±1.1^a^	0.68±0.10	7.5±1.65^a^	0.23±0.07^b^
SC	3.9±1.0^a^	0.70±0.09	10.0±2.8^a^	0.42±0.10
GA	6.2±1.4	0.79±0.13	13.3±3.9	0.35±0.10
GL	6.1±1.5	0.80±0.11	12.6±3.8	0.32±0.19
IA	7.7±2.3	0.80±0.16	16.0±2.5	0.60±0.14
IL	7.7±2.8	0.84±0.09	17.0±1.9	0.58±0.10
Nov	9.1±2.8	0.96±0.35	20.0±4.4	0.72±0.18
Hum	8.8±3.4	0.98±0.27	20.0±3.7	0.75±0.15

Data are presented as the mean ± SD values.

a P<0.05 was considered to indicate a statistically significant difference compared to all groups with the exception of LC and SC;

b P<0.05 was considered to indicate a statistically significant difference compared to all groups. CSII, continuous subcutaneous insulin infusion; LC group, long-term use of CSII therapy (insulinization); SC group, short-term use of CSII therapy; GA group, glargine + aspart intensive treatment; GL group, glargine + lyspro intensive treatment; IA group, insulin aspart 30 three times a day; IL group, insulin lispro 25 three times a day; Nov group, Novolin 30R two times a day; Hum group, Humulin 70/30 two times a day.

**Table III.** Results of management in each group.

Group	Ratio of patients not requiring OADs (%)	Time course of disease after termination of OADs (years)	Incidence of hypoglycemia (%)	Ratio of actual weight/standard weight
LC	13/30 (43.3%)^a^	2.5±0.7	6.7	1.3±0.35
SC	5/30 (16.7%)	3.0±1.1	13.3	1.0±0.45
GA	3/15 (20%)	2.2±0.6	26.7	1.2±0.41
GL	4/15 (26.7%)	2.5±0.3	26.7	1.2±0.30
IA	2/15 (13.3%)	2.7±0.7	53.3	1.2±0.36
IL	2/15 (13.3%)	2.9±0.5	46.7	1.1±0.38
Nov	1/15 (6.7%)	2.5±0.1	46.7	1.1±0.26
Hum	1/15 (6.7%)	2.1±0.3	53.3	1.2±0.35
GA+GL	7/30 (23.3%)			
IA+IL	4/30 (13.3%)			
Nov+Hum	2/30 (6.7%)			

Time course and weight ratios are presented as the mean ± SD.

a P<0.05 was considered to indicate statistically significant differences compared with the GA, GL, IA, IL, Nov or Hum groups. CSII, continuous subcutaneous insulin infusion; LC group, long-term use of CSII therapy (insulinization); SC group, short-term use of CSII therapy; GA group, glargine + aspart intensive treatment; GL group, glargine + lyspro intensive treatment; IA group, insulin aspart 30 three times a day; IL group, insulin lispro 25 three times a day; Nov group, Novolin 30R two times a day; Hum group, Humulin 70/30 two times a day.

**Table IV.** Comparison of HbA1c and β-cell function before and after treatment in each group.

Group	HbA1c (%)		Fasting plasma insulin (uIU/ml)	
	Before treatment	After treatment	Before treatment	After treatment
LC	12±2.8	5.7±0.2^a^	5.5±2.3	13±1.2^a,b^
SC	11±1.7	7.0±0.1	5.3±2.6	8.5±1.1
GA	12±2.3	6.4±0.3^a^	4.9±2.9	11±0.7^a^
GL	12±2.9	6.2±0.2^a^	5.1±2.2	11±0.6^a^
IA	11±1.3	7.2±0.4	5.2±1.9	7±1.1
IL	12±2.5	7.0±0.2	5.3±2.5	7±1.7
Nov	12±2.4	7.6±0.5	5.7±2.2	6±1.7
Hum	12±2.2	7.5±0.4	5.5±2.8	6±2.0

Data are presented as the mean ± SD.

a P<0.05 vs. SC, IA, IL, Nov or Hum groups;

b P<0.05 vs. GA or GL groups. LC group, long-term use of CSII therapy (insulinization); SC group, short-term use of CSII therapy; GA group, glargine + aspart intensive treatment; GL group, glargine + lyspro intensive treatment; IA group, insulin aspart 30 three times a day. IL group, Insulin lispro 25 three times a day; Nov group, Novolin 30R two times a day; Hum group, Humulin 70/30 two times a day.
